# An iterative expanding and shrinking process for processor allocation in mixed-parallel workflow scheduling

**DOI:** 10.1186/s40064-016-2808-y

**Published:** 2016-07-20

**Authors:** Kuo-Chan Huang, Wei-Ya Wu, Feng-Jian Wang, Hsiao-Ching Liu, Chun-Hao Hung

**Affiliations:** Department of Computer Science, National Chiao-Tung University, No. 1001, Ta-Hsueh Road, Hsinchu, Taiwan; Department of Computer Science, National Taichung University of Education, No. 140, Min-Shen Road, Taichung, Taiwan

**Keywords:** Workflow scheduling, Mixed parallelism, Moldable task, Processor allocation

## Abstract

Parallel computation has been widely applied in a variety of large-scale scientific and engineering applications. Many studies indicate that exploiting both task and data parallelisms, i.e. mixed-parallel workflows, to solve large computational problems can get better efficacy compared with either pure task parallelism or pure data parallelism. Scheduling traditional workflows of pure task parallelism on parallel systems has long been known to be an NP-complete problem. Mixed-parallel workflow scheduling has to deal with an additional challenging issue of processor allocation. In this paper, we explore the processor allocation issue in scheduling mixed-parallel workflows of moldable tasks, called M-task, and propose an Iterative Allocation Expanding and Shrinking (IAES) approach. Compared to previous approaches, our IAES has two distinguishing features. The first is allocating more processors to the tasks on allocated critical paths for effectively reducing the makespan of workflow execution. The second is allowing the processor allocation of an M-task to shrink during the iterative procedure, resulting in a more flexible and effective process for finding better allocation. The proposed IAES approach has been evaluated with a series of simulation experiments and compared to several well-known previous methods, including CPR, CPA, MCPA, and MCPA2. The experimental results indicate that our IAES approach outperforms those previous methods significantly in most situations, especially when nodes of the same layer in a workflow might have unequal workloads.

## Background

**Fig. 1:**
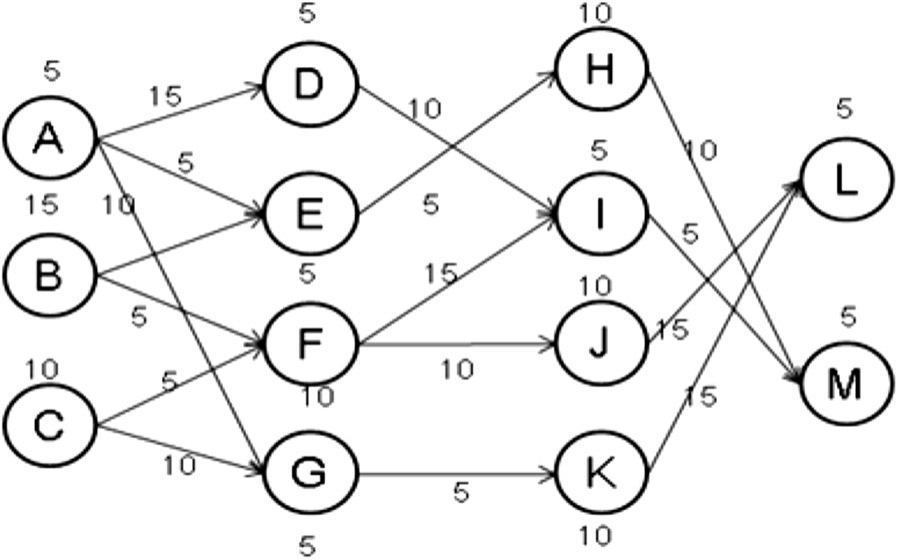
Task parallelism represented by a workflow

Parallel processing (Konstantopoulos 2015) has been applied to many computation demanding applications, especially a variety of large-scale scientific and engineering applications (Feitelson et al. 1997). In general, parallelism inherent in applications can be broadly divided into two types: data parallelism and task parallelism. For applications with data parallelism, usually a single program is executed on several processors simultaneously and each processor is responsible for processing a specific portion of data. Many tools and programming libraries have been developed to aid writing parallel programs with data parallelism, such as MPI (Quinn 2008), OpenMP (Chapman and Jost 2007), and OpenCL (Munshi et al. 2011). The computational structure of an application with task parallelism usually can be represented by a Directed-Acyclic-Graph (DAG) (Topcuoglu et al. 2002; Ramaswamy et al. 1997) based task dependency graph, commonly called a workflow, and looks like Fig. 1. Each node represents a task which usually executes a specific program. The number next to each node indicates the computation workload of the task. Based on the computation workload and processor speed, the required execution time of a task on a processor can be derived. The edges represent the dependence between tasks and the number next to an edge means the amount of data to transfer between two tasks. The required data transmission time depends on the amount of data and the communication bandwidth between the processors running the two tasks. A scheduler has to schedule and allocate each task according to the dependence specified in the workflow. Scheduling is an important and challenging research field (Severino et al. 2014; Amirghasemi and Zamani 2014), and scheduling such kind of workflows on parallel systems has long been known to be a NP-complete problem (Pinedo 2008). Therefore, many heuristic methods have been proposed to produce efficient schedules within a reasonable time period (Topcuoglu et al. 2002; Ramaswamy et al. 1997; Radulescu et al. 2001; Radulescu and van Gemund 2001; Bansal et al. 2006; N’Takṕe et al. 2007; Yu and Shi 2009).

As applications become even more complex and computation demanding, recently many studies indicate that exploiting both task and data parallelism can be a promising approach to getting better efficacy compared with either pure task parallelism or pure data parallelism models (Hsu et al. 2011). The computational structure exploiting both task and data parallelism is sometimes called a mixed-parallel model (N’Takṕe et al. 2007), which means that each node in Fig. 1 can itself be a parallel program exploiting data parallelism (Feitelson et al. 1997). Scheduling mixed-parallel workflows is more complicated than dealing with simple task-parallel workflows since each task might require more than one processor for execution, and therefore the resource fragmentation issue in scheduling data-parallel jobs also has to be considered for producing efficient schedules (Hsu et al. 2011).

There is a particular class of mixed-parallel workflows where each data-parallel task in a workflow is moldable (Feitelson et al. 1997). A moldable job is a kind of data-parallel jobs which can be executed with an arbitrary number of processors depending on resource availability (Feitelson et al. 1997). Such moldable jobs in the mixed-parallel workflows are called M-task in the literature (Radulescu et al. 2001; Radulescu and van Gemund 2001). Scheduling mixed-parallel workflows of M-tasks is even more challenging because it usually involves two different kinds of activities, *allocation* and *mapping*, where the allocation activities are not needed in scheduling other types of workflows. The allocation activities are for determining an appropriate amount of processors to be allocated for each M-task. The mapping activities regards mapping each M-task onto the processors in a parallel system to form a temporal and spatial schedule of the entire mixed-parallel workflow.

This paper aims at developing an effective processor allocation approach for M-tasks in order to improve the overall execution performance of mixed-parallel workflows. In general, the goal of processor allocation for M-tasks is concerned about critical path reduction and allocation fragmentation avoidance. Most of previous approaches adjust the allocation of each M-task in a monotonically increasing manner until a predefined scheduling criterion is reached in the iterative process. In this paper, we propose an Iterative Allocation Expanding and Shrinking (IAES) approach to dealing with the above two concerns. IAES has two distinct features compared to existing methods. The first one is that IAES allows the allocation of an M-task to shrink during the iterative procedure, leading to a more flexible and effective processor allocation process. Secondly, IAES adopts a more accurate mechanism based on the temporarily scheduled Earliest-Start-Time (EST) and Earliest-Finish-Time (EFT) of each M-task to avoid possible processor allocation fragmentation. Based on these two features, IAES has potential to outperform existing methods. The proposed IAES approach has been evaluated with a series of simulation experiments using both workflow structures of real world applications and synthetic workflows generated by the widely used approach in (Topcuoglu et al. 2002). The performance results demonstrate that IAES outperforms existing methods in most situations in terms of average makespan and average SLR.

The remainder of this paper is organized as follows. Section two discusses “Related work” on workflow scheduling, including task-parallel and mixed-parallel workflows. Section “Processor allocation for M-tasks in mixed-parallel workflows” presents our IAES approach and illustrates how it could outperform existing methods. Section “Performance evaluation and discussion” presents the experimental results and discussions. Section “Conclusions and future work” concludes the paper.

## Related work

Most previous research works on workflow scheduling deal with task-parallel workflows, where each task in a workflow is a serial job requiring only one processor for execution. The taxonomy proposed in (Yu et al. 2010) classifies such workflow scheduling algorithms into two groups: heuristics-based and meta-heuristics-based, and further, heuristics-based scheduling algorithms fall into several categories, including (1) immediate task scheduling, (2) list-based scheduling, (3) cluster-based scheduling, and (4) duplication-based scheduling.

Immediate task scheduling is the simplest heuristic for workflow applications. It makes schedule decisions based on the availability of tasks only. One typical example is the Myopic algorithm (Sakellariou et al. 2005), which has been implemented in some Grid systems such as Condor DAGMan (Tannenbaum et al. 2002). A list-based scheduling algorithm comprises two phases: the task prioritizing phase and the resource selection phase. The task prioritizing phase sets the priority of each task and generates a scheduling list by sorting the tasks according to their priorities. Then, the resource selection phase picks tasks from the list in order and maps each task to a most appropriate resource for it. List-based heuristics (Topcuoglu et al. 2002; Kwok and Ahmad 1996; Wu and Gajski 1990) received the most attention because of their simplicity and flexibility. For example, HEFT (Topcuoglu et al. 2002) is a well-known list-based workflow scheduling algorithm for heterogeneous environments. It first traverses a workflow from the exit node to the entry node in order to calculate an upward rank value for each task. The tasks are then sorted in non-ascending order of their ranks. According to the order, each task is assigned to the resource that minimizes its Earliest Finish Time (EFT). Many heuristics have been developed based on HEFT (Yu and Shi 2009; Bittencourt et al. 2010; Ghanem et al. 2010).

Both cluster-based heuristics and duplication-based heuristics are designed to reduce the communication costs between interdependent tasks (Yang and Gerasoulis 1994; Darbha and Agrawal 1998; Park et al. 1997; Bajaj and Agrawal 2004). In cluster-based heuristics, several tasks with data dependency are put into the same group (cluster) first, and then are assigned onto the same resource for communication cost reduction. On the other hand, duplicated-based heuristics try to reduce the communication cost for a task to transmit data to the resource of its succeeding task(s) through duplicating the task on the destination processors. Duplication-based heuristics were shown potential to achieve good performance when scheduling a single workflow (Park et al. 1997). However, they might not be appropriate when scheduling multiple concurrent workflows since task duplication in a workflow would consume extra computation resources and thus degrade the performance of other workflows.

The meta-heuristics-based approaches provide both a general structure and strategy guidelines for developing a heuristic to fit a particular kind of problem. Meta-heuristics-based algorithms, generally applied to large and complicated problems, provide an efficient way of moving quickly toward a very good solution, although not optimal. There are in general three kinds of meta-heuristics-based approaches based on Greedy Randomized Adaptive Search Procedure (GRASP) (Resende and Ribeiro 2002), Genetic Algorithm (Singh and Youssef 1996), and Simulated Annealing (YarKhan and Dongarra 2002). There are comparisons (Tannenbaum et al. 2002; Blythe et al. 2005) between the heuristics-based approaches and meta-heuristics-based approaches. The comparison shows that meta-heuristics-based approaches usually perform better than heuristics-based approaches, since a meta-heuristics-based method has more chance to approach the globally optimal solution than heuristics-based methods. However, the scheduling time in meta-heuristics-based algorithms is significantly higher than heuristics-based algorithms, and the time complexity of the meta-heuristics based algorithms grows more rapidly than that of the heuristics-based algorithms if the size of workflows become larger.

As workflow applications become more complex and computation-demanding, mixed-parallel workflow computing (N’Takṕe et al. 2007) becomes a promising and important computing model where each task in a workflow might be a data-parallel program requiring multiple processors for execution. Many studies have shown that mixed-parallel computation achieves better performance compared to either pure data parallelism or pure task parallelism (Ramaswamy et al. 1997; Radulescu et al. 2001; Radulescu and van Gemund 2001; Hunold 2010). According to (Feitelson et al. 1997) data-parallel jobs usually can be classified into four categories: rigid, moldable, malleable, and evolving. The work on mixed-parallel workflow scheduling in (Hsu et al. 2011) deals with the case that each data-parallel task within the workflow is rigid which means that each data-parallel task comes with a pre-specified number of processors to use and the scheduler has to allocate exactly that amount of processors to the task. On the other hand, in Radulescu et al. (2001), Radulescu and van Gemund (2001), N’Takṕe et al. (2007) and some other research works, the data-parallel tasks are assumed to be moldable and the focus is on how to determine a most appropriate number of processors to use for each moldable task, M-task, within a mixed-parallel workflow. This is also the research issue to be dealt with in this paper.

For mixed-parallel workflows of M-tasks, according to how allocation and mapping activities are arranged during the scheduling process, existing scheduling approaches in the literature can be broadly divided into two categories: one step and two steps. One-step approaches produce the schedule in an iterative manner. Each scheduling iteration consists of two steps where the first step adjusts the allocation of each M-task and the second step maps all M-tasks onto processors to check whether an improved schedule is achieved or not. The feedback of the second step will then guide the next iteration’s first step. A typical example of one-step approaches is CPR (Radulescu et al. 2001), which is a greedy iterative algorithm. At first, the algorithm assigns one processor for each M-task and computes the resultant makespan based on the list-scheduling approach. Then, an iterative procedure is applied to increase the number of assigned processors for each M-task until the entire workflow’s makespan cannot be improved further.

To reduce scheduling overhead, many two-step approaches have been proposed in the literature, such as TSAS (Ramaswamy et al. 1997), CPA (Radulescu and van Gemund 2001), MCPA (Bansal et al. 2006), and MCPA2 (Hunold 2010). In two-step approaches, the iterative process is only applied to the allocation step, which determines the most appropriate allocation of each M-task simply based on the static structural property of the workflow to be scheduled. Then, the mapping step decides the spatial and temporal assignment of each M-task onto the parallel computing platforms to produce the workflow execution schedule according to the allocation result in the first step. CPA (Radulescu and van Gemund 2001) is one of the most famous two-step algorithms. Many later two-step algorithms were developed based on its critical path strategy, such as MCPA (Bansal et al. 2006), MCPA2 (Hunold 2010). They differ in how to decide the allocation limit of each M-task.

## Processor allocation for M-tasks in mixed-parallel workflows

**Fig. 2:**
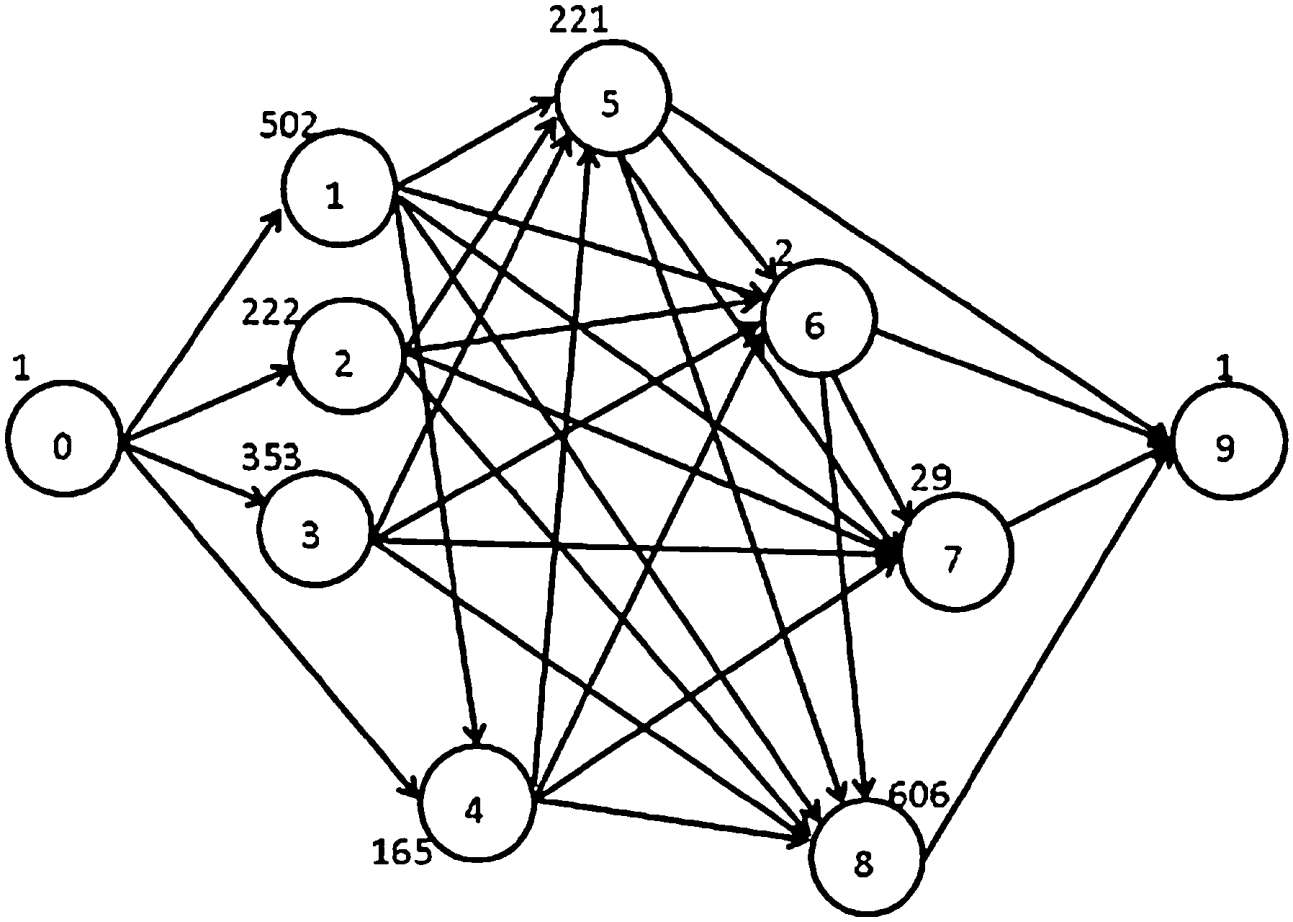
An example mixed-parallel workflow

In this section, we explore the issues of processor allocation for M-tasks when scheduling mixed-parallel workflows, discuss the pros and cons of previous methods, and then propose a new Iterative Allocation Expanding and Shrinking (IAES) approach to the processor allocation problem. We use an example mixed-parallel workflow, shown in Fig. 2, to illustrate the characteristics of each method and demonstrate the superiority of our IAES approach. Each node in Fig. 2 represents an M-task with its ID shown in the circle, and the number next to each node is the computation workload of the corresponding M-task.

### Workflow model

As in most of the literatures (Topcuoglu et al. 2002; Ramaswamy et al. 1997; Radulescu et al. 2001; Radulescu and van Gemund 2001; Bansal et al. 2006), we assume that a mixed-parallel workflow application of moldable jobs can be modeled as a Directed Acyclic Graph (DAG), e.g. Figure 2, to represent the constituent tasks and their execution order. The DAG is defined as a pair (*V*, *E*), where *V* and *E* are finite sets. $V = \{ t_{i} |i = 1, \ldots , n\}$ denotes the set of n nodes representing the constituent data-parallel tasks, each of which is a moldable job (Feitelson et al. 1997) and can be executed with an arbitrary number of processors depending on resource availability. *E* denotes the set of edges $\{ e_{i, j} |1 \le i, j \le n\}$ where $e_{i, j}$, is an arc from $t_{i}$ to $t_{j}$, representing that $t_{j}$ can only be executed after $t_{i}$ finishes its computation due to the control or data dependency between them. $t_{i}$ is thus usually called the parent of $t_{j}$. A task without ancestor is called an entry task and a task without any descendant is an exit task. It is assumed that there is only one entry task and one exit task in a workflow application.

Each node in the task graph is called an M-task (Radulescu and van Gemund 2001) since it is moldable and can run with an arbitrary number of processors. Each node is annotated with the computation workload of the corresponding M-task. The required computation time of an M-task with a specific number of processors can be obtained either by user estimation or by applications’ speedup models (Ramaswamy et al. 1997; Rauber and Rünger 1998). In our study, the execution time of an M-task with different number of processors is calculated by Amdahl’s law (Kleinrock and Huang 1992), and the fraction of workload that must be executed serially within an M-task is assumed to be 0.2. A task can be executed only when it receives all the required data from its parents. The data transfer between two tasks incurs a communication cost that depends on network capabilities. In traditional research works on task-parallel workflows (Prasanna et al. 1994; Kwok and Ahmad 1996; Wu and Gajski 1990), the communication cost between two tasks is assumed to be negligible if these two tasks are allocated on the same processor. Therefore, reducing inter-task communication costs becomes an important part when scheduling task-parallel workflows. However, for mixed-parallel workflows, since each M-task might use a different number of processors for execution, there is always data communication or redistribution costs between two connected tasks. Therefore, in this paper we focus on the processor allocation issues of M-tasks and ignore the data communication costs.

### Common notations and terms used in M-task allocation algorithms

Before elaborating on the M-task allocation methods, we first introduce several key notations and terms (Sinnen 2007) as follows, which will be used in describing the M-task allocation algorithms.

- *P* The number of processors in a parallel computing system.
- *Schedule* A schedule determines the spatial and temporal assignment of tasks in a DAG to processors. A schedule is usually generated by a specific scheduling algorithm on a specific number of processors.
- *np*(*t*) The number of processors allocated to task *t*.
- $T_{w} \left( {t , np\left( t \right)} \right)$ The computation cost of a node *t*, representing the required computation time of the corresponding M-task with *np*(*t*) processors.
- *Path length* The length of a path is the summation of the computation cost of each node on the path. Since we don’t consider data communication costs in the study as explained in the previous section, the path length defined here excludes the communication costs between nodes on the path.
- *Allocated path length* Based on a schedule, the allocated path length is defined to be the finish time of the last node on the path subtracted by the start time of the first node on the path.
- *tl*(*n*) The top level of a node *n* in a DAG, which is the length of the longest path ending in *n*, but excludes the computation cost of *n*.
- *bl*(*n*) The bottom level of a node *n* in a DAG, which is the length of the longest path starting with *n*.
- *Schedule length* The length of a schedule is the finish time of the exit task on it, assuming the entry task starts at time zero.
- *Critical path* It is a longest path in a DAG. The critical path gains its importance for workflow scheduling from the fact that its length is a lower bound for the schedule length.
- *Allocated critical path* The path of the longest allocated path length in a schedule.
- *Critical tasks* The nodes on critical paths or allocated critical paths, which are of particular importance in the following M-task allocation methods.
- *MLS* M-task list scheduling, which is a procedure applying simple list scheduling to produce the execution schedule of a workflow on a parallel system of a specific number of processors after the number of allocated processors for each M-task is known (Radulescu et al. 2001). This procedure can provide the estimated execution time, i.e. makespan, of a workflow.
- *Makespan* The total execution time for a workflow application. It is used to measure the performance of a scheduling algorithm from the perspective of workflow applications. However, makespan usually varies widely among workflows with different sizes and other properties.
- *Schedule Length Ratio (SLR)* The ratio of a workflow’s makespan over the length of its critical path. SLR tries to measure the performance of scheduling algorithms regardless of the variation in workflow’s size. In the experiments, the length of the critical path is calculated by assuming each M-task runs with only one processor.

### Previous methods

This section presents several most well-known processor allocation methods for M-tasks in mixed-parallel workflows and discusses their pros and cons.

#### CPA

One of the most famous methods for scheduling mixed-parallel workflows of M-tasks is the Critical Path and Allocation (CPA) algorithm (Radulescu and van Gemund 2001). It continues to increase the number of processors, starting from one, for each task on the critical path while the condition, $T_{CP} > T_{A}$, holds, where

$$ T_{CP} = max_{t \in V} \left\{ {bl\left( t \right)} \right\}\quad {\text{and}}\quad T_{A} = \frac{1}{P}\mathop \sum \limits_{t \in V} \left( {T_{w} \left( {t, np\left( t \right)} \right) \times np\left( t \right)} \right). $$

Both *T*_*CP*_ and *T*_*A*_ represent theoretical lower bounds for a workflow’s makespan, but characterize two different aspects. $T_{CP}$ is a measure of the dependence paths, that can be shortened by increasing the number of processors for the tasks on the critical paths. On the other hand, $T_{A}$ is a measure of processor utilization, which would become larger when allocating more processors to tasks. The detailed algorithm of CPA is shown in Algorithm 1


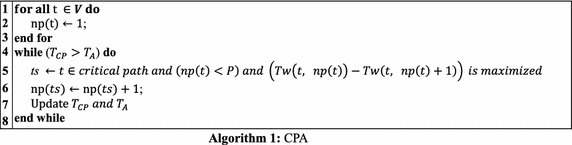


**Fig. 3:**
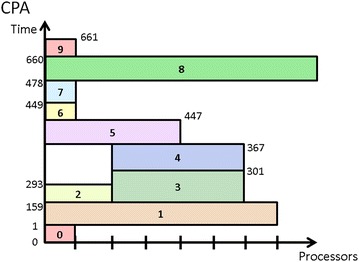
Schedule generated by CPA

CPA is in general efficient. However, since CPA allocates processors to tasks at a per task basis, in many cases, it might lead to unnecessary resource fragmentation and wasting because the total allocated processors of concurrent tasks exceed the system’s capacity. Figure 3 is the schedule generated by CPA for the mixed-parallel workflow in Fig. 2 and shows an example for such situation. As shown in Fig. 2, *t*_1_, *t*_2_, and *t*_3_ are three concurrent tasks at the same level and can be run in parallel to exploit task parallelism. However, in the schedule generated by CPA, as shown in Fig. 3, *t*_1_, *t*_2_, and *t*_3_ do not run in parallel since the total number of processors allocated to these three tasks is more than the available number of processors in the system. Therefore, the potential task parallelism among tasks *t*_1_, *t*_2_, and *t*_3_ is deteriorated which leads to increased makespan of the workflow and reduced resource utilization rate of the system.

#### MCPA

The Modified Critical Path and Area-based (MPCA) algorithm (Bansal et al. 2006) was developed based on improving the processor allocation phase of CPA, which aims to make better processor allocation for data-parallel tasks without sacrificing the essential task parallelism available in the workflow applications. MCPA divides the tasks of a workflow into different layers according to their dependency relationship. Thus, potential task parallelism within a workflow comes from the tasks at the same layer, which can run concurrently. MCPA bounds the number of processors that can be allocated to each layer’s tasks by the system’s capacity. The detailed algorithm of MCPA is shown in Algorithm 2.


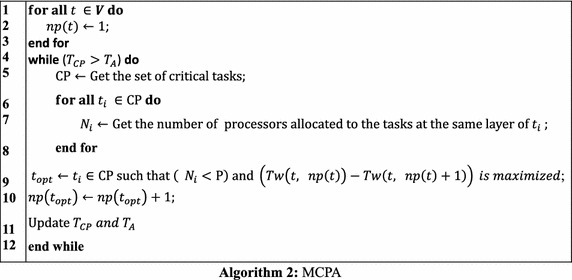


**Fig. 4:**
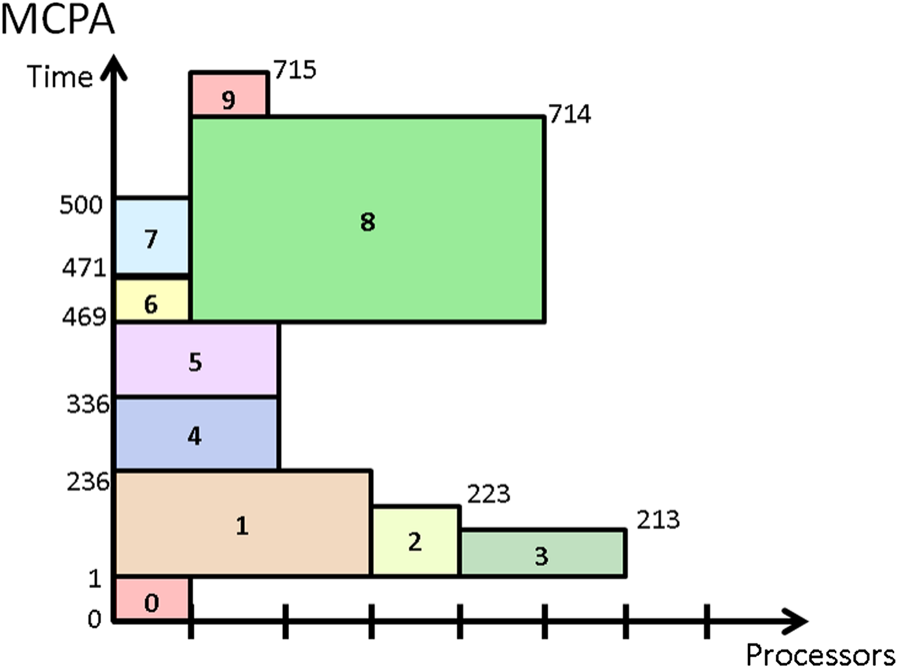
Schedule generated by MCPA

Figure 4 shows the schedule generated by MCPA for the same mixed-parallel workflow. In contrast to the schedule in Fig. 3, tasks *t*_1_, *t*_2_, and *t*_3_ are now running in parallel, demonstrating MCPA’s advantage of retaining task parallelism among tasks at the same layer (Bansal et al. 2006). However, the makespan in Fig. 4 is worse than that in Fig. 3, indicating the drawback of MCPA (Hunold 2010) that it fails to deliver efficient schedules for irregular workflows where concurrent tasks differ significantly in the computation costs or there are more concurrent tasks than processors in the system.

#### MCPA2

MCPA2 (Hunold 2010) was proposed to overcome the drawbacks of CPA and MCPA. The detailed approach of MPCA2 is shown in the following Algorithm 3. We first define a set of specific notations as follows, which are used in the algorithm description (Hunold 2010).

- pl(*v*): precedence level of node *v*.
- ${\text{DFS}}\_{\text{DEPTH}}\left( v \right)$: depth of node *v* determined by a depth-first search procedure.
- prec_alloc(*l*): number of processors allocated to tasks at precedence layer *l*.
- PL: set of precedence levels.
- prec_p(*l*): bound for number of processors at layer *l*.
- lp(*v*): nodes in the same precedence layer as *v*.
- *wr*: a scaling factor of *P* defined by users in order to loosen the restrictions when allocating processors; 0 < *wr* ≤ 1.
- W(*v*): work area, i.e. the product of np(*v*) and $Tw\left( {v,   np\left( v \right)} \right)$, when executing *v*.
- $h_{\varvec{t}}^{{\varvec{min}}}$: minimum height of the precedence layer of *t*.
- *cr*_*min*_: a minimum cover ratio defined by users.

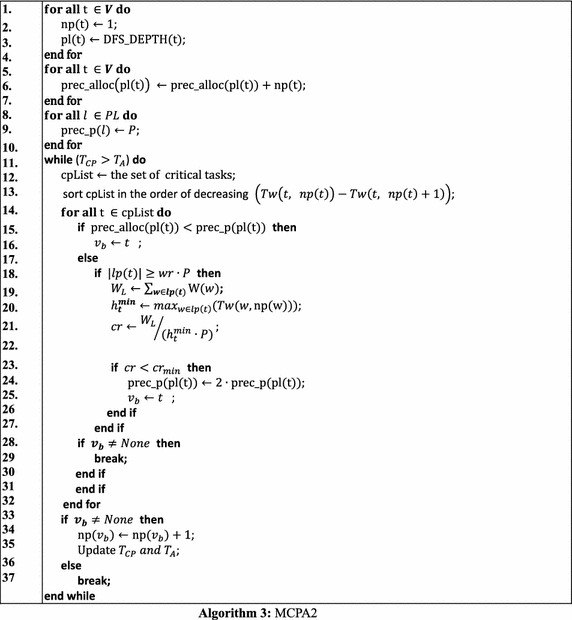


**Fig. 5:**
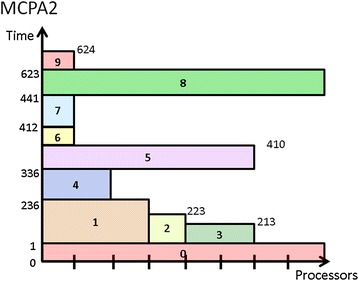
Schedule generated by MCPA2

The main idea of MCPA2 is that it would allow more processors to be allocated to tasks on the critical paths, called *critical tasks*, even though that would lead to a situation where the total allocated processors of the tasks at the same layer would exceed the system’s capacity. Therefore, the most important part of the algorithm is to define a variable *cr* that denotes the *cover ratio* of a layer which is the sum of works done by all tasks of a layer divided by the minimum height of the layer. The works done by a layer, *L*, of tasks is defined by $$ W_{L} = \sum\nolimits_{v \in L} {W\left( v \right)} $$ and the minimum height of a layer is $$ L_{A} = h_{t}^{min} \cdot P $$. Based on these two variables, the *cover ratio* is given by $$ cr = {W_{L} }/{L_{A} }$$. Figure 5 shows that MCPA2 has the potential to outperform CPA and MCPA2, compared to Figs. 3 and 4.

#### CPR

**Fig. 6:**
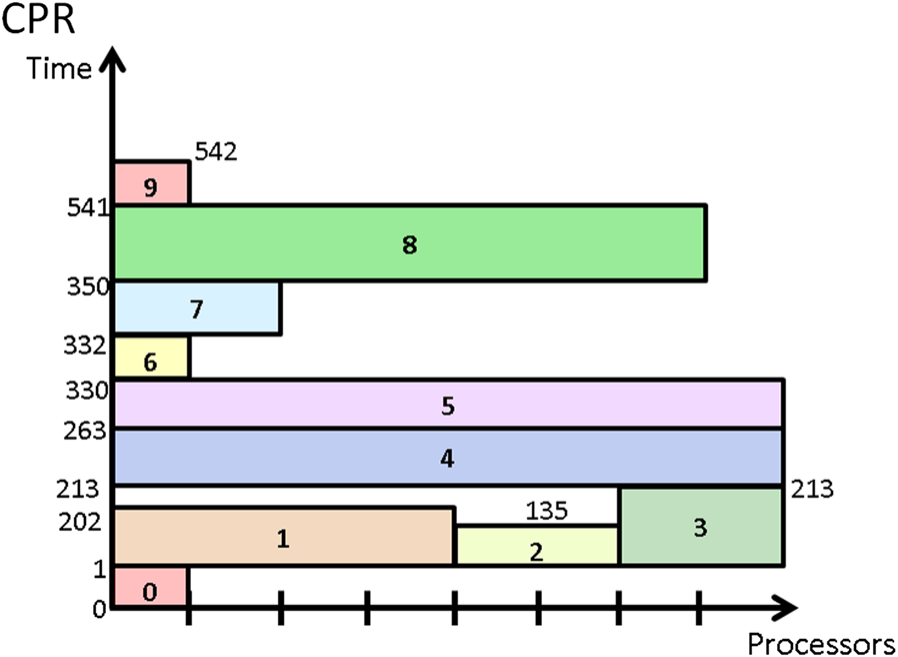
Schedule generated by CPR


The above three approaches, CPA, MCPA, MCPA2, are well known two-step approaches for scheduling mixed-parallel workflows of M-tasks. They can quickly produce a schedule but at the cost of schedule efficiency. On the other hand, the Critical Path Reduction (CPR) (Radulescu et al. 2001) approach is a one-step algorithm that can deliver more efficient schedules than two-step approaches through an iterative process of M-task allocation and M-task list scheduling (MLS for short), while leading to a longer algorithm computation time. At each iteration, CPR increases the number of processors allocated to a particular M-task and then tests whether the execution time of the entire workflow decreases through the MLS procedure. CPR commits such an allocation increment only if the execution time decreases. The iterative process of CPR stops when there is no task for which increasing the allocated processor number can reduce the workflow execution time further. Algorithm 4 shows the detailed operations of CPR. Figure 6 shows the schedule generated by CPR, demonstrating it outperforms the previous three two-step approaches in terms of makespan.

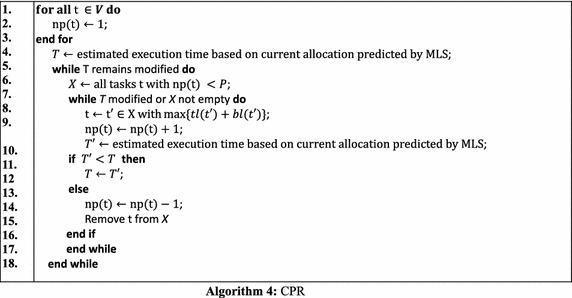


### An Iterative Allocation Expanding and Shrinking approach

In the following, we present an Iterative Allocation Expanding and Shrinking (IAES) approach to the processor allocation problem when scheduling mixed-parallel workflows of M-tasks. IAES is a one-step approach and has two distinguishing features compared to previous approaches. The first is reducing the lengths of allocated critical paths (Sinnen 2007) instead of the static critical paths in workflows. The second is allowing to shrink the number of processors allocated to an M-task during the iterative process, while most previous approaches adopt non-decreasing M-task allocation mechanisms.

**Fig. 7:**
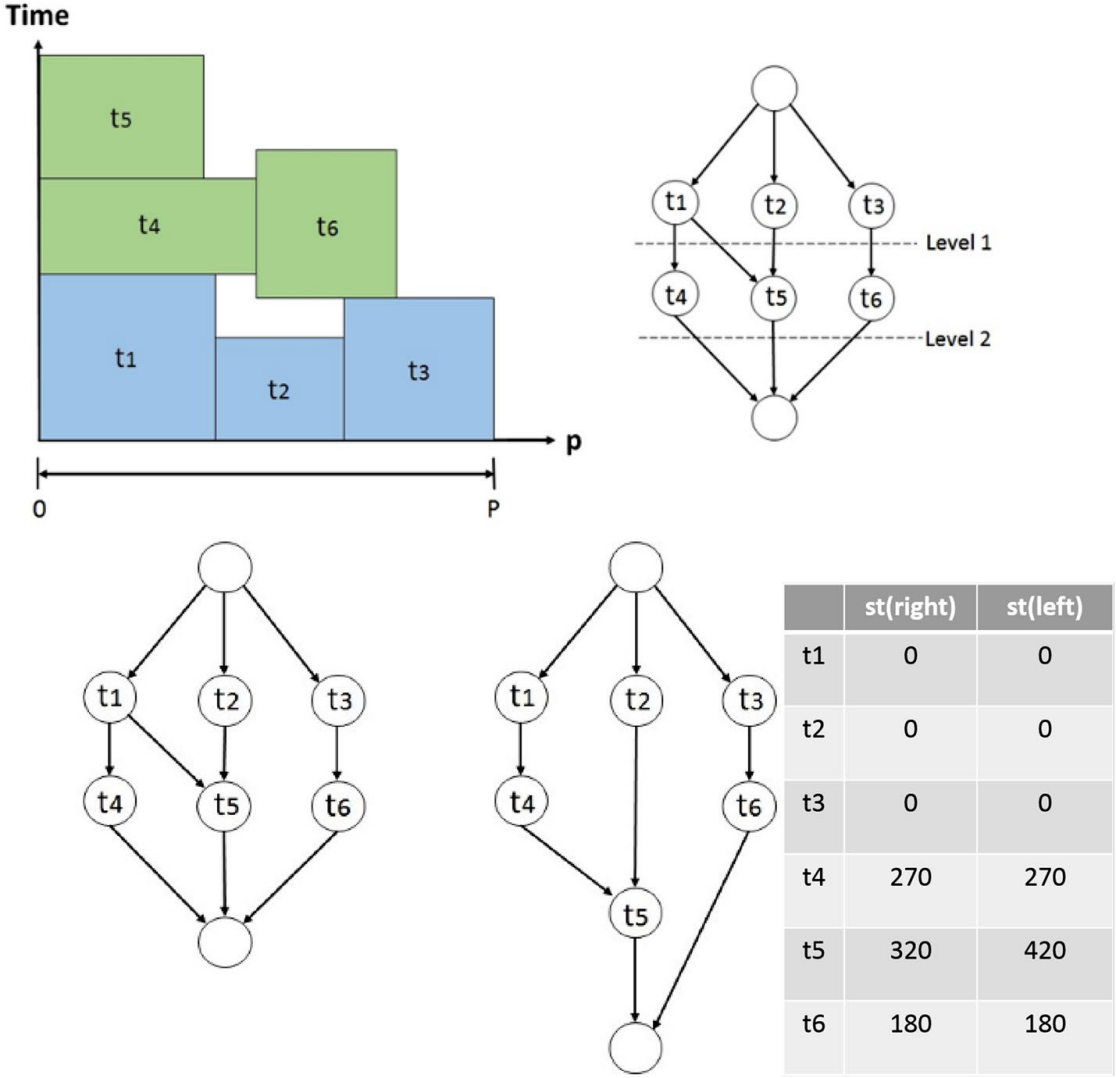
Allocated critical path

Previous one-step and two-step approaches aim to decrease the length of critical paths in the M-task allocation phase. Most of them determine the critical paths based on the original static properties of DAGs. However, due to the limitation of available processors, tasks might not start immediately once becoming ready and therefore the critical path in the final schedule, called allocated critical path (Sinnen 2007), might be different from the one in the DAG. Figure 7 shows such an example, where the lower left part is the original DAG and the lower right part is the DAG modified according to the schedule, shown in the upper part, to reflect the allocated critical path t_1_ → t_4_ → t_5_. Although task 5 can run concurrent to task 4 according to the original DAG structure, in the schedule task 5 has to run after task 4 due to the limitation of system capacity. Therefore, the critical path changes. Increasing the processor allocation of tasks on the static critical path might not improve the makespan of the entire workflow execution. Our IAES increases the processor allocation of tasks on the allocated critical paths which can effectively reduce the required workflow execution time.

**Fig. 8:**
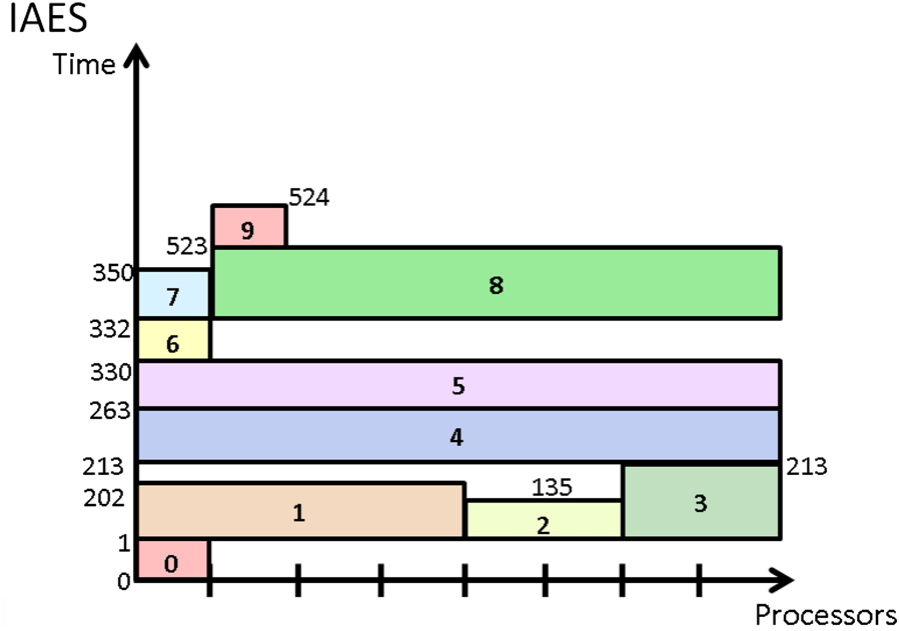
Schedule generated by IAES


IAES allows the processor allocation of an M-task to shrink during the iterative procedure, leading to a more flexible and effective process which is promising in finding better schedules. The detailed approach of IAES is shown in Algorithm 5. The algorithm starts with allocating one processor to each task. Then, it calculates the makespan of the entire workflow execution with this allocation (lines 1–3). Next, the algorithm iteratively increases or shrinks the number of processors allocated to each task until the resultant makespan remains unchanged after an iteration (lines 5–36). The distinguishing shrinking process in IAES is described in lines 18–30 which is applied when the expanding of a critical task results in worse makespan. The shrinking process first find tasks which might be affected by the allocation expansion of the critical task, i.e. whose execution periods overlap the time period between the expanded task’s start time and finish time. Then, it tries to shrink some of those tasks’ allocation to check whether an improved schedule can be achieved. Figure 8 shows the schedule produced by IAES, which achieves the shortest makespan among all the methods discussed in this section, demonstrating the superiority of IAES over CPA, MCPA, MCPA2, and CPR.

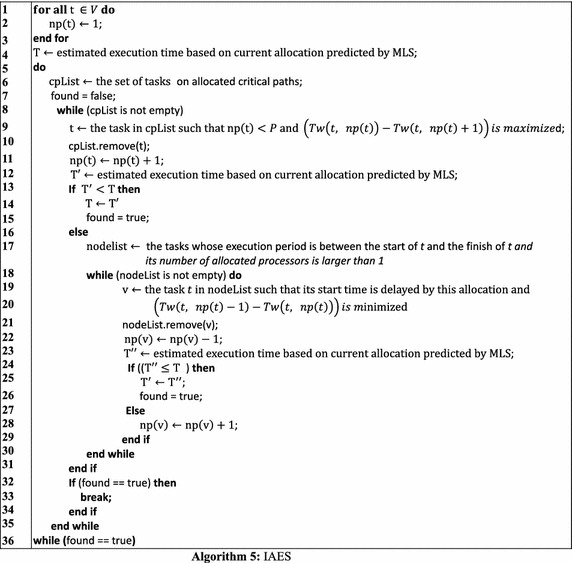


## Performance evaluation and discussion

This section evaluates the proposed IAES approach and compares it to several well-known previous algorithms discussed in section “Processor allocation for m-tasks in mixed-parallel workflows” with a series of simulation experiments. Section “Experimental setup and performance metrics” introduces the setup for the following experiments and the metrics used in the performance analysis. Section “Experimental results” presents and compare the experimental results.

### Experimental setup and performance metrics

The experiments were conducted on a software simulator developed by ourselves in C++ based on the discrete-event simulation methodology (Fishman 2001). The simulator maintains the task interdependence in each workflow and calls the chosen algorithm to schedule the workflows. To make thorough performance evaluation, like in most related works (Radulescu et al. 2001; Radulescu and van Gemund 2001; Bansal et al. 2006; Hunold 2010), we conducted various experiments of different configurations, e.g. different workflow structures, different number of processors, and different number of nodes within a workflow. For workflow structures, we experimented with two real world applications and synthetic workflows. The structures of synthetic workflows were generated using the approach described in (Topcuoglu et al. 2002), which has been widely used in many research works of workflow scheduling. In the following experiments, the execution time of an M-task with different number of processors is calculated by Amdahl’s law (Kleinrock and Huang 1992) as follows,

$$ w\left( {t, np\left( t \right)} \right) = \left( {\alpha + \frac{1 - \alpha }{ np\left( t \right)}} \right)\tau , $$

where $\tau$ is the task’s execution time on a single processor, $\alpha$ is the fraction of workload that must be executed serially and was set to 0.2. The performance metrics used in the experiments are described below. In each experiment, the average values of 30 runs with different workflows in terms of makspan and SLR, respectively, are used to evaluate different methods.

### Experimental results

**Fig. 9:**
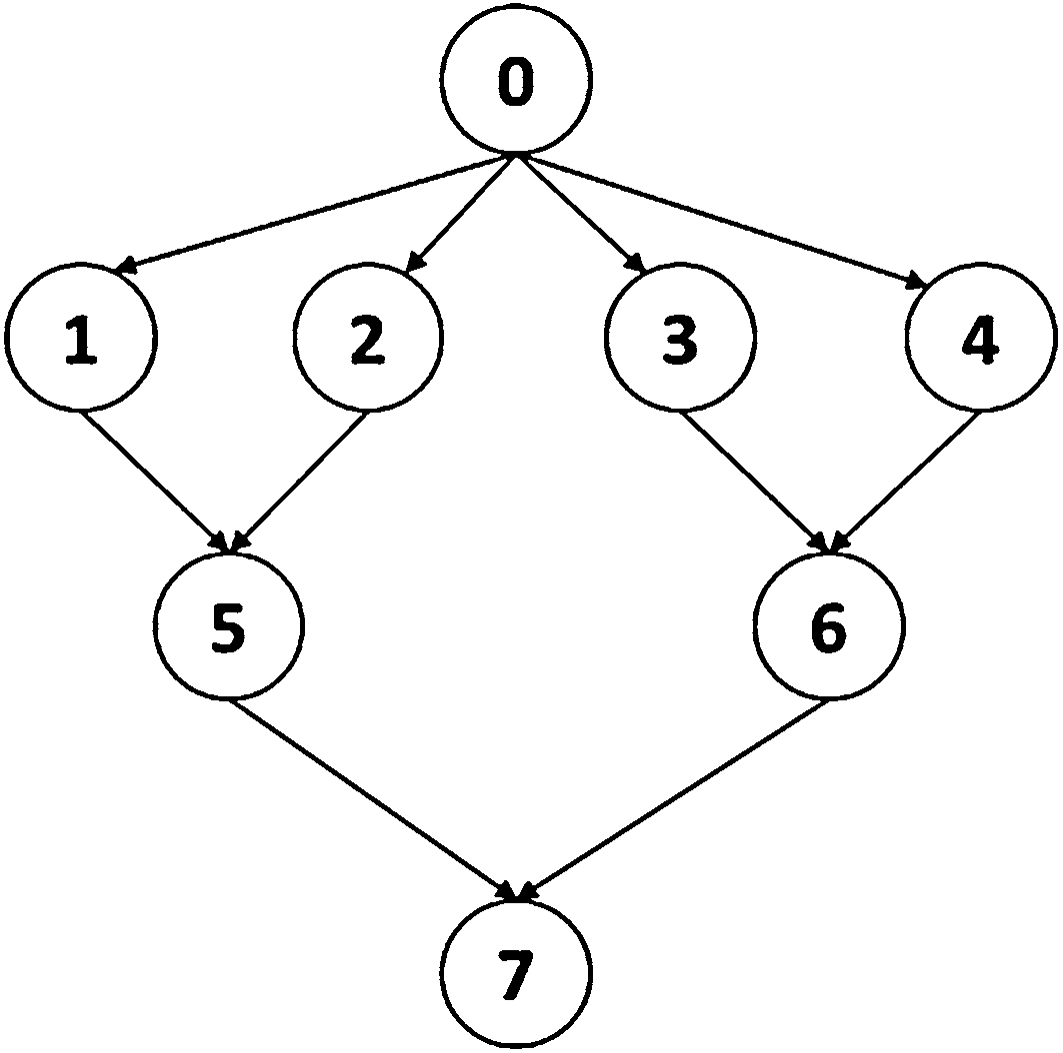
Workflow structure of Matmul

This section presents the experimental results comparing our IAES with CPR (Radulescu et al. 2001), CPA (Radulescu and van Gemund 2001), MCPA (Bansal et al. 2006), and MCPA2 (Hunold 2010). Figure 9 is the workflow structure of a real world application, Matrix Multiplication (Matmul), which has been used in the experiments of many research works on mixed-parallel workflow scheduling, such as (Radulescu et al. 2001; Radulescu and van Gemund 2001; Bansal et al. 2006).

Tables 1, 2, 3 and 4 present performance evaluation of the five M-task allocation methods using the real world workflow structure in Fig. 9. The italic and underlined numbers in the tables indicate the best performance in each experiment. To make thorough performance evaluation, we conducted two types of experiments. In the experiments of Tables 1 and 2, tasks of the same layer in the workflow are assumed to have equal workloads, while unequal workloads are assumed in the experiments of Tables 3 and 4. A task’s workload is the amount of work to compute. Based on the workload and processor speed, the required execution time of a task on a processor can be derived. Since in our experiments, the processors are assumed to be homogeneous, *equal workload* implies the same execution time and *unequal workload* indicates different execution time. Unequal-workload cases were also studied in (Hunold 2010) where the term *irregular* was used. There are real applications corresponding to the unequal-workload cases in our experiments, such as sparse matrix computation and other irregular computational problems. In both types of experiments, we evaluated the M-task allocation methods across parallel computer systems of four different numbers of processors, i.e. 8, 16, 32, and 64. Tables 1 and 3 show the performance comparison in terms of average makespan and Tables 2 and 4 present the performance evaluation in terms of average SLR.

**Table 1:** Average makespan (s) for Matmul structure of equal workloads

	np = 8	np = 16	np = 32	np = 64
CPA	188,162	136,645	111,903	78,397
MCPA	*117,603*	*84,003*	*67,203*	*58,802*
MCPA2	*117,603*	*84,003*	*67,203*	*58,802*
CPR	*117,603*	*84,003*	*67,203*	*58,802*
IAES	*117,603*	*84,003*	*67,203*	*58,802*

**Table 2:** Average SLR for Matmul structure of equal workloads

	np = 8	np = 16	np = 32	np = 64
CPA	0.30	0.21	0.18	0.12
MCPA	*0.18*	*0.13*	*0.11*	*0.09*
MCPA2	*0.18*	*0.13*	*0.11*	*0.09*
CPR	*0.18*	*0.13*	*0.11*	*0.09*
IAES	*0.18*	*0.13*	*0.11*	*0.09*

**Table 3:** Average makespan (s) for Matmul structure of unequal workloads

	np = 8	np = 16	np = 32	np = 64
CPA	17,301	13,217	11,480	11,132
MCPA	13,857	10,006	8299	7642
MCPA2	16,351	10,410	8835	8329
CPR	14,064	10,920	9433	8788
IAES	*13,280*	*9583*	*7888*	*7351*

**Table 4:** Average SLR for Matmul structure of unequal workloads

	np = 8	np = 16	np = 32	np = 64
CPA	0.31	0.24	0.21	0.20
MCPA	0.25	0.18	0.15	0.14
MCPA2	0.29	0.19	0.16	0.15
CPR	0.25	0.20	0.17	0.16
IAES	*0.24*	*0.17*	*0.15*	*0.13*

The experimental results in Tables 1 and 2 show that for the workflow structure of Matmul, MCPA, MCPA2, CPR, and our IAES achieve the same performance when nodes of the same layer have equal workloads. On the other hand, Tables 3 and 4 indicate that the five M-task allocation methods lead to significantly different performance when nodes of the same layer might have unequal workloads. In both types of experiments, our IAES can achieve the best performance, while CPA performs the worst because it allocates processors to tasks at a per task basis and thus leads to unnecessary resource fragmentation and wasting. The experimental results in Tables 3 and 4 indicate that the number of processors might influence the relative performance of the M-task allocation methods. For example, CPR outperforms MCPA2 when the system has eight processors, while MCPA2 achieves better performance than CPR for systems of more processors.

**Fig. 10:**
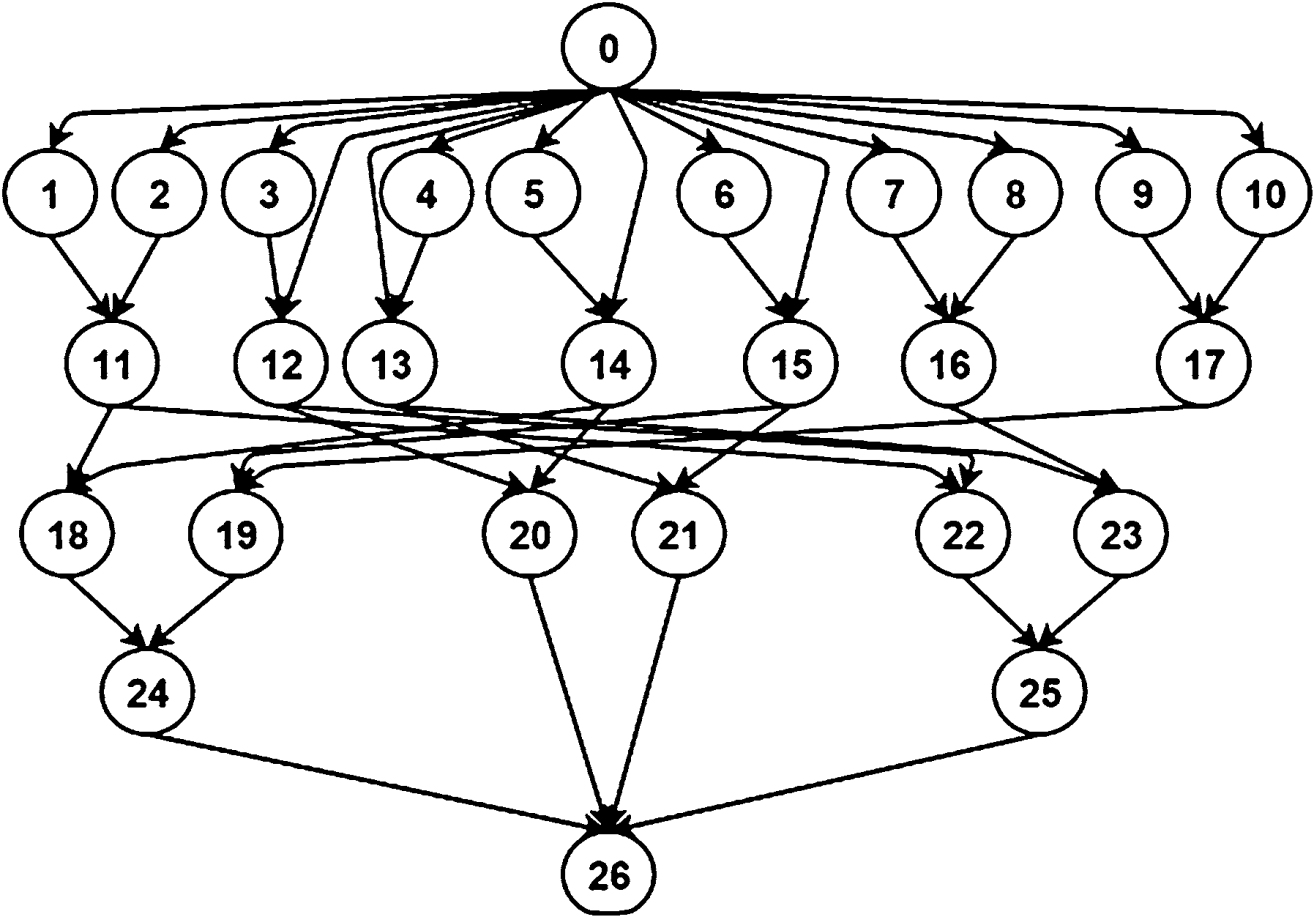
Workflow structure of Strassen

Figure 10 is the workflow structure of another real world application, Strassen Matrix Multiplication (Strassen), which has also been used in the experiments of many research works on mixed-parallel workflow scheduling, including (Radulescu et al. 2001; Radulescu and van Gemund 2001; Bansal et al. 2006).

Tables 5, 6, 7 and 8 present the performance evaluation using the real world workflow structure of Strassen in Fig. 10. In the experiments of equal workloads for nodes of the same layer, as shown in Tables 5 and 6, MCPA2 outperforms the others when the system has only eight processors, while CPR achieves the best performance for systems of more processors, e.g. 16, 32, or 64 processors. CPR has the potential to outperform other two-step algorithms because its one-step nature can deliver more efficient schedules due to tight integration of M-task allocation and scheduling at the cost of longer algorithm execution time. For the experiments in Tables 7 and 8 where nodes of the same layer might have unequal workloads, our IAES outperforms all the other methods across systems of different number of processors, while the relative performance of the other four methods varies when the number of processors changes. Our IAES has the potential to outperform all other methods for the cases of unequal workloads because it allows to shrink the number of processors allocated to an M-task during the iterative process, leading to more flexible schedules.

**Table 5:** Average makespan (s) for Strassen structure of equal workloads

	np = 8	np = 16	np = 32	np = 64
CPA	33,888	20,723	15,153	12,587
MCPA	39,202	17,844	14,544	13,985
MCPA2	*30,244*	19,826	13,275	10,841
CPR	31,363	*15,456*	*11,100*	*8940*
IAES	32,483	20,444	12,506	9984

**Table 6:** Average SLR for Strassen structure of equal workloads

	np = 8	np = 16	np = 32	np = 64
CPA	0.45	0.28	0.20	0.17
MCPA	0.52	0.24	0.19	0.19
MCPA2	*0.40*	0.27	0.18	0.15
CPR	0.42	*0.21*	*0.15*	*0.12*
IAES	0.43	0.27	0.17	0.13

**Table 7:** Average makespan (s) for Strassen structure of unequal workloads

	np = 8	np = 16	np = 32	np = 64
CPA	47,882	28,738	20,614	18,996
MCPA	45,577	27,320	19,208	19,041
MCPA2	47,682	28,658	19,748	17,952
CPR	49,590	26,893	18,717	16,233
IAES	*43,775*	*26,681*	*17,593*	*15,608*

**Table 8:** Average SLR for Strassen structure of unequal workloads

	np = 8	np = 16	np = 32	np = 64
CPA	0.60	0.38	0.27	0.22
MCPA	0.57	0.37	0.26	0.22
MCPA2	0.60	0.38	0.27	0.21
CPR	0.63	*0.35*	0.25	0.19
IAES	*0.55*	*0.35*	*0.24*	*0.18*

The following presents the experimental results with synthetic workflow structures generated by the widely used approach described in (Topcuoglu et al. 2002). We used the following parameters to generate different workflow structures.

- Node: the number of nodes in a workflow.
- Shape: a number controlling the shape of a workflow. A higher shape value results in a shorter workflow with a higher parallelism degree. Otherwise, a longer workflow with a lower parallelism degree is generated. Shape is randomly selected from the set {0.5, 1.0, 2.0}.
- OutDegree: the maximum number of immediate descendants of a task. OutDegree is randomly selected from the set {1, 2, 3, 4, 5}.

Each experiment was repeated 30 times with different randomly generated workflows and the average performance values are presented in the following tables. Tables 9, 10, 11 and 12 show the experimental results across systems of different numbers of processors, where each workflow contains ten nodes, but might have different structure. In the experiments of Tables 9 and 10 where nodes of the same layer have equal workloads, our IAES outperforms the other methods in most situations except the system of eight processors, where CPR achieves the best performance. When nodes of the same layer might have unequal workloads, as in Tables 11 and 12, our IAES outperforms all the other methods across systems of different number of processors, while the relative strength of the other four methods might be different when the number of processors changes.

**Table 9:** Average makespan (s) for synthetic workflows of nodes with equal workloads

	np = 8	np = 16	np = 32	np = 64
CPA	43,932	34,206	28,855	26,166
MCPA	44,658	25,712	18,960	15,569
MCPA2	42,623	27,291	20,688	17,398
CPR	*27,537*	20,879	18,165	16,810
IAES	30,606	*20,134*	*16,116*	*14,243*

**Table 10:** Average SLR for synthetic workflows of nodes with equal workloads

	np = 8	np = 16	np = 32	np = 64
CPA	0.42	0.33	0.28	0.25
MCPA	0.42	0.24	0.18	0.15
MCPA2	0.41	0.26	0.20	0.17
CPR	*0.27*	0.20	0.17	0.16
IAES	0.30	*0.19*	*0.15*	*0.14*

**Table 11:** Average makespan (s) for synthetic workflows of nodes with unequal workloads

	np = 8	np = 16	np = 32	np = 64
CPA	23,687	18,618	16,086	15,139
MCPA	27,295	16,600	12,664	10,839
MCPA2	23,696	17,712	13,789	11,995
CPR	19,285	14,135	12,496	11,814
IAES	*18,507*	*13,322*	*11,107*	*10,155*

**Table 12:** Average SLR for synthetic workflows of nodes with unequal workloads

	np = 8	np = 16	np = 32	np = 64
CPA	0.33	0.26	0.23	0.21
MCPA	0.37	0.23	0.17	0.15
MCPA2	0.33	0.24	0.19	0.17
CPR	0.27	0.20	0.17	0.16
IAES	*0.26*	*0.19*	*0.15*	*0.14*

Tables 13, 14, 15 and 16 present experiments across workflows of four different numbers of nodes, i.e. 30, 40, 50, and 60. In the experiments, workflows were scheduled onto a parallel system of 64 processors. Tables 13 and 14 show performance results of the experiments where nodes of the same layer in the workflow are assumed to have equal workloads. Tables 15 and 16 are for experiments where nodes of the same layer might have unequal workloads. Our IAES outperforms the other methods significantly in all the experiments, while the relative performance of the other four methods varies when the number of processors changes.

**Table 13:** Average makespan (s) for synthetic workflows of nodes with equal workloads

	30 nodes	40 nodes	50 nodes	60 nodes
CPA	39,209	44,023	49,117	68,521
MCPA	30,496	43,750	48,942	73,699
MCPA2	35,115	39,740	48,655	54,891
CPR	46,649	54,030	56,507	66,616
IAES	*28,481*	*33,991*	*38,508*	*48,018*

**Table 14:** Average SLR for synthetic workflows of nodes with equal workloads

	30 nodes	40 nodes	50 nodes	60 nodes
CPA	0.27	0.25	0.28	0.32
MCPA	0.21	0.25	0.28	0.34
MCPA2	0.25	0.23	0.28	0.26
CPR	0.32	0.30	0.32	0.31
IAES	*0.20*	*0.20*	*0.23*	*0.23*

**Table 15:** Average makespan (s) for synthetic workflows of nodes with unequal workloads

	30 nodes	40 nodes	50 nodes	60 nodes
CPA	21,229	22,829	25,162	26,118
MCPA	19,166	22,717	23,861	28,512
MCPA2	20,704	22,337	23,999	25,879
CPR	27,211	27,923	27,179	35,546
IAES	*18,314*	*21,088*	*21,757*	*24,035*

**Table 16:** Average SLR for synthetic workflows of nodes with unequal workloads

	30 nodes	40 nodes	50 nodes	60 nodes
CPA	0.22	0.23	0.25	0.25
MCPA	0.20	*0.22*	0.23	0.28
MCPA2	0.21	*0.22*	0.23	0.25
CPR	0.27	0.28	0.28	0.33
IAES	*0.19*	*0.22*	*0.21*	*0.23*

In summary, among all the M-task allocation methods evaluated, our IAES achieves the best performance in most situations. For very simple and regular workflow structure of few nodes, e.g. Matmul in Fig. 9, most M-task allocation methods might achieve similar or even the same performance as shown in Tables 1 and 2. When nodes of the same layer in a workflow have equal workloads, CPR might have advantage over other methods for specific kinds of workflow structure, e.g. Strassen in Fig. 10, as shown in Tables 5 and 6. On the other hand, our IAES has superiority over the other methods when nodes of the same layer in a workflow might have unequal workloads, consistently achieving the best performance across different workflow structures, different numbers of nodes, and different numbers of processors, as shown in the experimental results.

Table 17 compares the execution overhead of different M-task allocation algorithms in terms of algorithm computation time for scheduling a workflow. The time shown in Table 17 is the average number of 30 runs with different workflows in the experiments. All the workflows used in the experiment of each method contain 30 nodes but have different structures. MCPA requires the least computation time because it enforces a limit on the total number of processors allocated to tasks at the same layer, leading to a smaller search space. Our IAES needs the longest computation time among all methods. However, the algorithm overhead is negligible, compared to the performance gain shown in the experimental results and the long execution time commonly seen for real world workflow applications.

**Table 17:** Algorithm computation time (s)

	CPA	MCPA	MCPA2	CPR	IAES
Time	0.0019	0.0015	0.0020	0.0101	0.0172

## Conclusions and future work

This paper presents our study on scheduling mixed-parallel workflows of moldable tasks, M-tasks, in parallel computing systems. We propose a new one-step algorithm, called Iterative Allocation Expending and Shrinking (IAES), which has two distinct features compared to existing methods. The first one is that IAES allows the allocation of an M-task to shrink during the iterative procedure, avoiding possible processor allocation fragmentation and making the scheduling process more flexible and effective for finding better schedules. Secondly, IAES adopts a dynamic mechanism to find critical tasks and allocate more processors to them based on the concept of allocated critical path, which can effectively reduce the makespan of workflow execution. Based on these two distinguishing features, our IAES outperforms well-known previous approaches, including CPA, MCPA, MCPA2, and CPR, significantly in a series of simulation experiments using both workflow structures of real world applications and synthetic workflows.

In this paper, we investigate mixed-parallel workflow scheduling for single workflow. A promising future research direction is to expand our research work to deal with scheduling online multiple mixed-parallel workflows, which is a common need in modern shared parallel computing environments.
